# Supplementary figures and images for: CRISPR editing of sftb-1/SF3B1 in Caenorhabditis elegans allows the identification of synthetic interactions with cancer-related mutations and the chemical inhibition of splicing
Source: PLoS Genet. 2019 Oct 21;15(10):e1008464. doi: 10.1371/journal.pgen.1008464 (PMC6830814; doi:10.1371/journal.pgen.1008464)

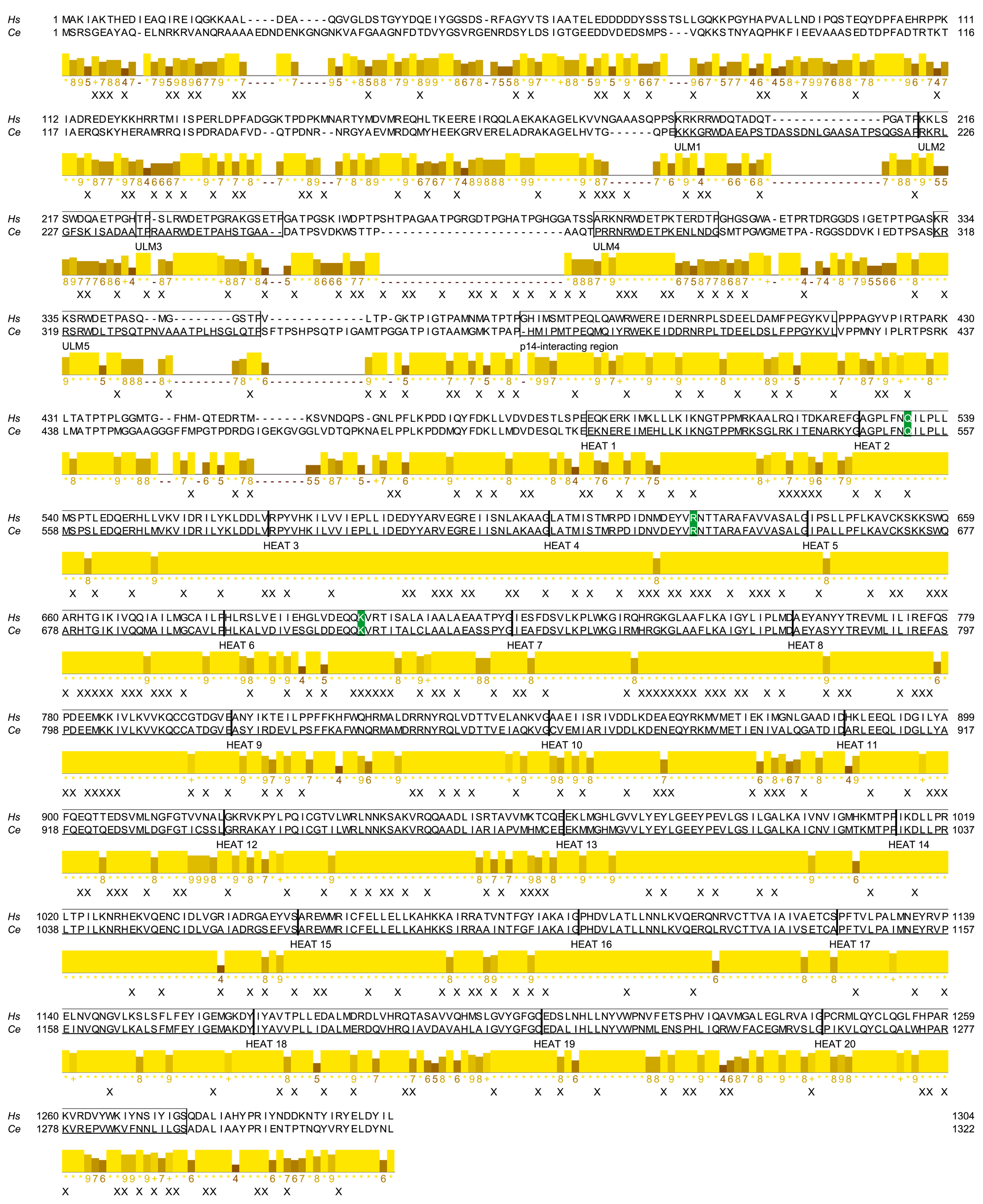

Supplement: S1 Fig — Protein sequence alignment of human SF3B1 (Hs) and worm SFTB-1 (Ce). The yellow histogram and the numerical index represent the degree of conservation (* corresponds to the maximum degree of conservation). U2AF ligand motifs (ULMs), p14-interacting region and HEAT domains are indicated in boxes. A cross symbol (X) below the conservation score indicates positions where missense mutations have been reported in human cancers (COSMIC database) at different frequencies. Residues that are relevant for this study are highlighted in green. (TIF) [file pgen.1008464.s001.tif]

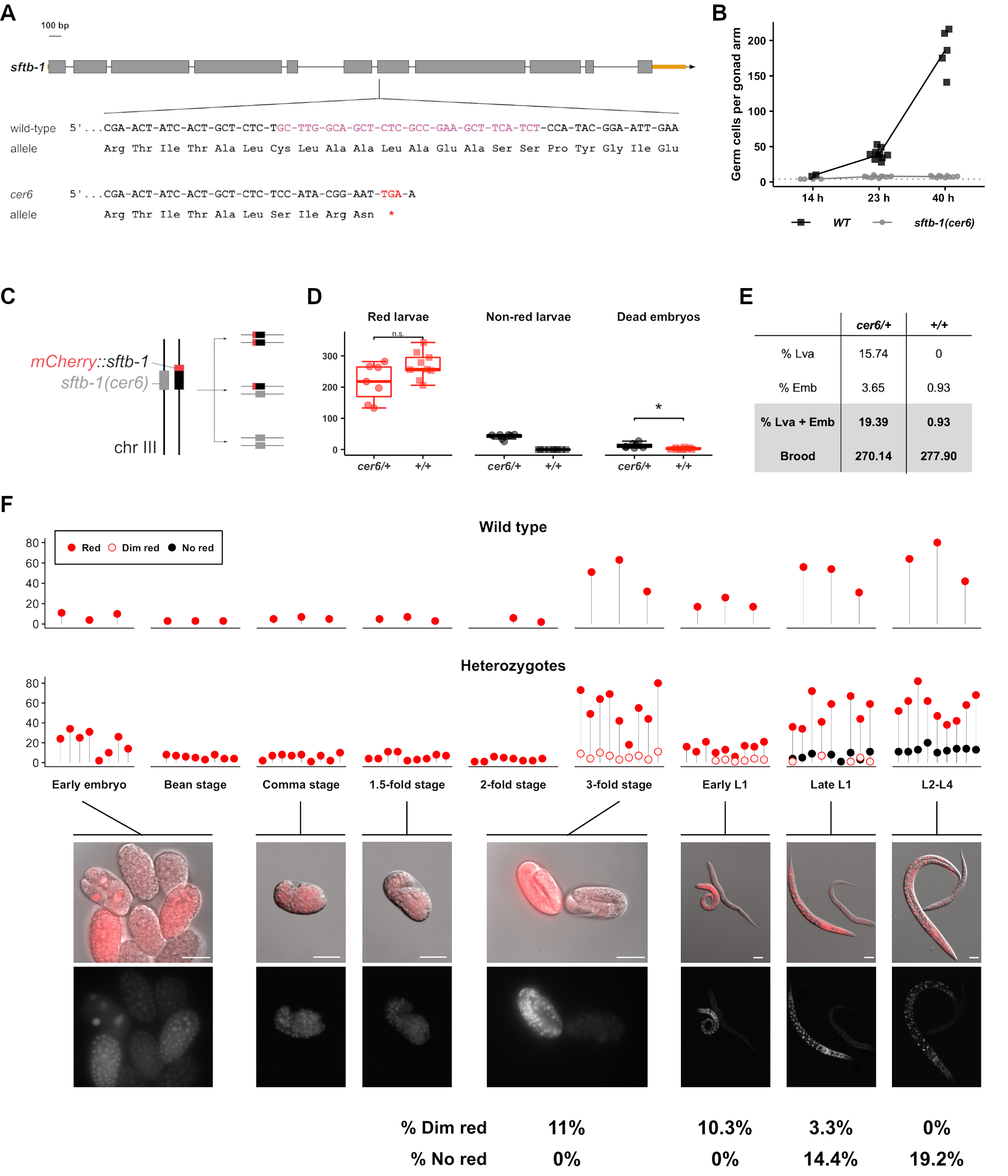

Supplement: S2 Fig — (A) Schematic representation of the sftb-1 locus. Gray boxes indicate exons, connecting lines indicate introns. Left and right orange boxes represent predicted 5’ and 3’ untranslated regions, respectively. In pink, 29 nucleotides that are deleted in the cer6 allele. Predicted amino acids resulting from translating the WT or cer6 alleles are shown below the DNA sequence. The frameshift caused by the deletion introduces a premature stop codon in the cer6 transcript (colored in red). Scale bar, 100 base pairs (bp). (B) Germ cell number of WT (N2 strain) or homozygous sftb-1(cer6) synchronized larvae grown for 14, 23, and 40 hours at 20°C. In WT worms, differentiating spermatocytes were not included at the last time point, and the number of germ cells was counted in one gonad arm only. Black and gray lines connect the median germ cell number at each time point in WT and sftb-1(cer6) worms, respectively. The dotted line represents the four germ cells that are normally present in recently hatched L1 larvae; these cells will resume proliferation during the L1 stage. Between 2 and 10 animals per condition were scored (N = 1). (C) Schematic representation of the compound heterozygous strain CER505: sftb-1(cer6)/sftb-1(cer114[mCherry∷sftb-1]) III. (D) Total number of progeny laid by heterozygous (cer6/+) or WT (+/+) worms from a CER505 population (n = 7, 9; N = 1). The number of F1 larvae expressing mCherry∷SFTB-1 was not significantly different between both groups (n.s.), while cer6/+ animals laid a significantly higher number of dead embryos which lacked red fluorescence (Mann-Whitney’s test; * p<0.05). Heterozygotes also segregated a number of non-red larvae that were arrested and were not observed in WT plates, indicating that they were cer6/cer6 animals. ‘+’ denotes the cer114[mCherry∷sftb-1] allele. Dots represent measures in individual worms, overlaid to Tukey-style boxplots. (E) Mean percentage of non-red F1 arrested larvae (% Lva), F1 dead embryos (% Emb), and mean b [file pgen.1008464.s002.tif]

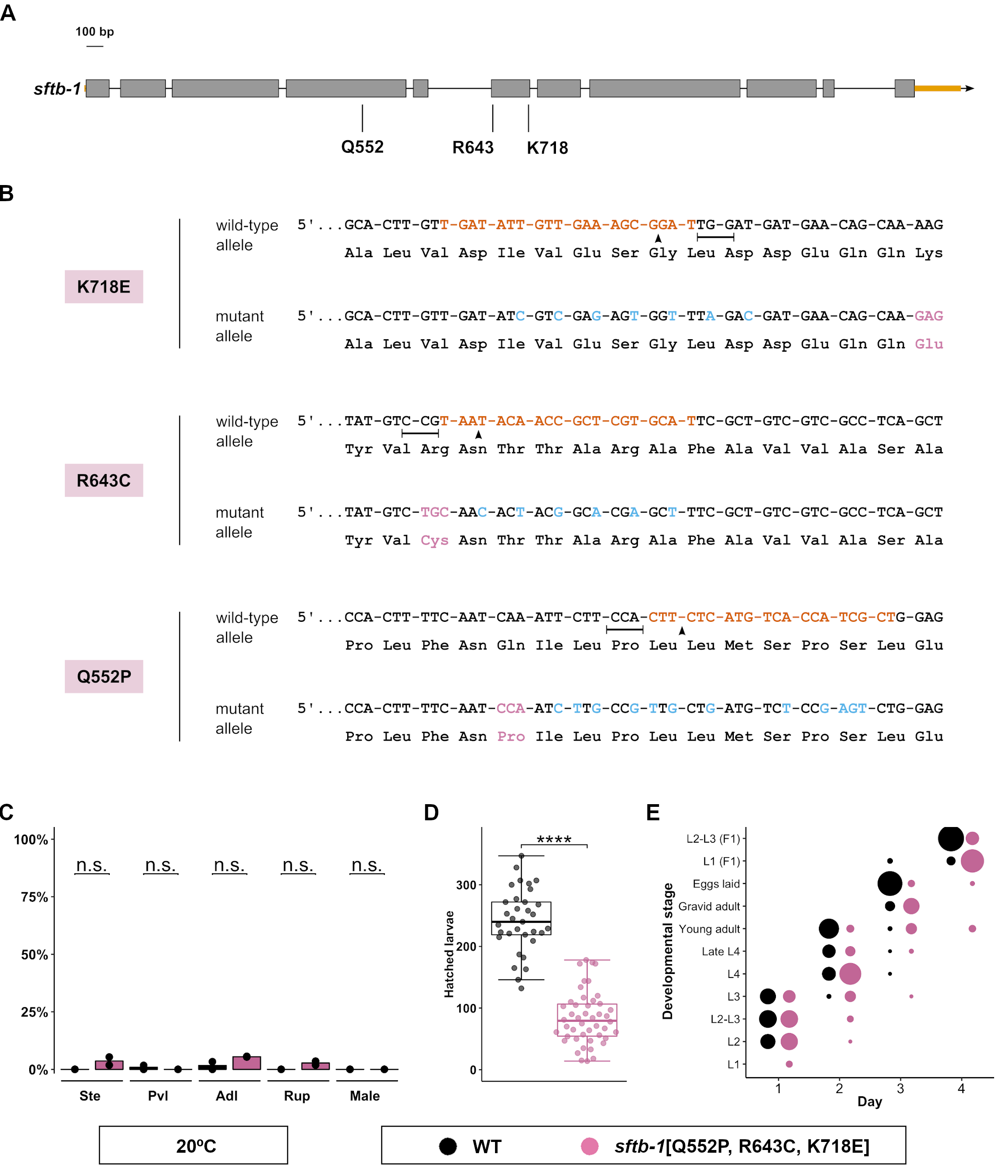

Supplement: S3 Fig — (A) Schematic representation of the sftb-1 locus, as in S2 Fig. The position of the triplets encoding the three mutated residues in this study (Q552, R643 and K718) is indicated. Scale bar, 100 bp. (B) Molecular details of the three sftb-1 missense mutations generated by CRISPR/Cas9. In WT alleles, the crRNA sequence used is indicated by orange nucleotides, the protospacer-adjacent motif (PAM) is underlined and the Cas9 cut site is indicated with a black arrowhead. In mutant alleles, synonymous mutations introduced to prevent partial recombination events and to improve primer specificity for mutant allele detection by PCR are indicated in blue. Mutated codons and the corresponding amino acids are colored in pink. (C) Bars represent mean incidence of different phenotypes observed in sftb-1[Q552P, R643C, K718E] worms at 20°C, while dots represent percentages observed in each replicate (n = 116, 109; N = 2). (D) Brood size of WT or sftb-1[Q552P, R643C, K718E] worms at 20°C (n = 33, 46; N = 2). Dots represent values for each individual animal, overlaid to Tukey-style boxplots. (E) sftb-1[Q552P, R643C, K718E] animals present a mild developmental delay at 20°C. Dot sizes represent the proportion of the population at each stage (n = 116, 109; N = 2). Statistics: (C), Fisher’s exact test (D), Student’s t-test (unpaired, two-tailed). n.s., no significant difference, **** p<0.0001. (TIF) [file pgen.1008464.s003.tif]

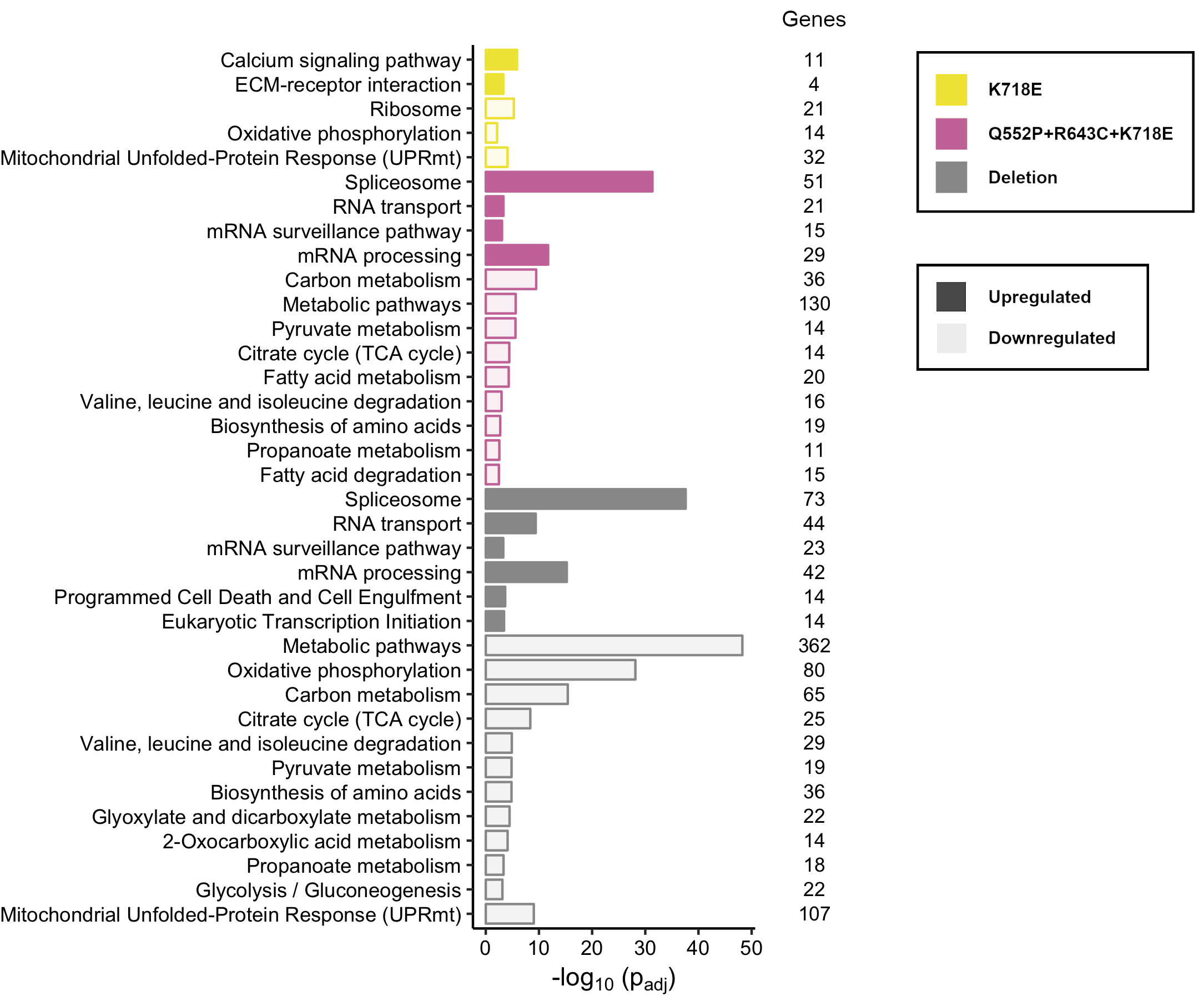

Supplement: S4 Fig — Significantly enriched functional terms in the distinct datasets are listed (padj<0.01). Bars represent the adjusted enrichment p-values in negative log10 scale, color-coded by mutant strain. Solid and clear bars denote enriched terms in upregulated and downregulated genes, respectively. The number of genes with differentially expressed transcripts belonging to each category is shown. The analysis was performed with the g:GOSt tool in g:Profiler, and only biological pathways from KEGG and WikiPathways databases are shown. (TIF) [file pgen.1008464.s004.tif]

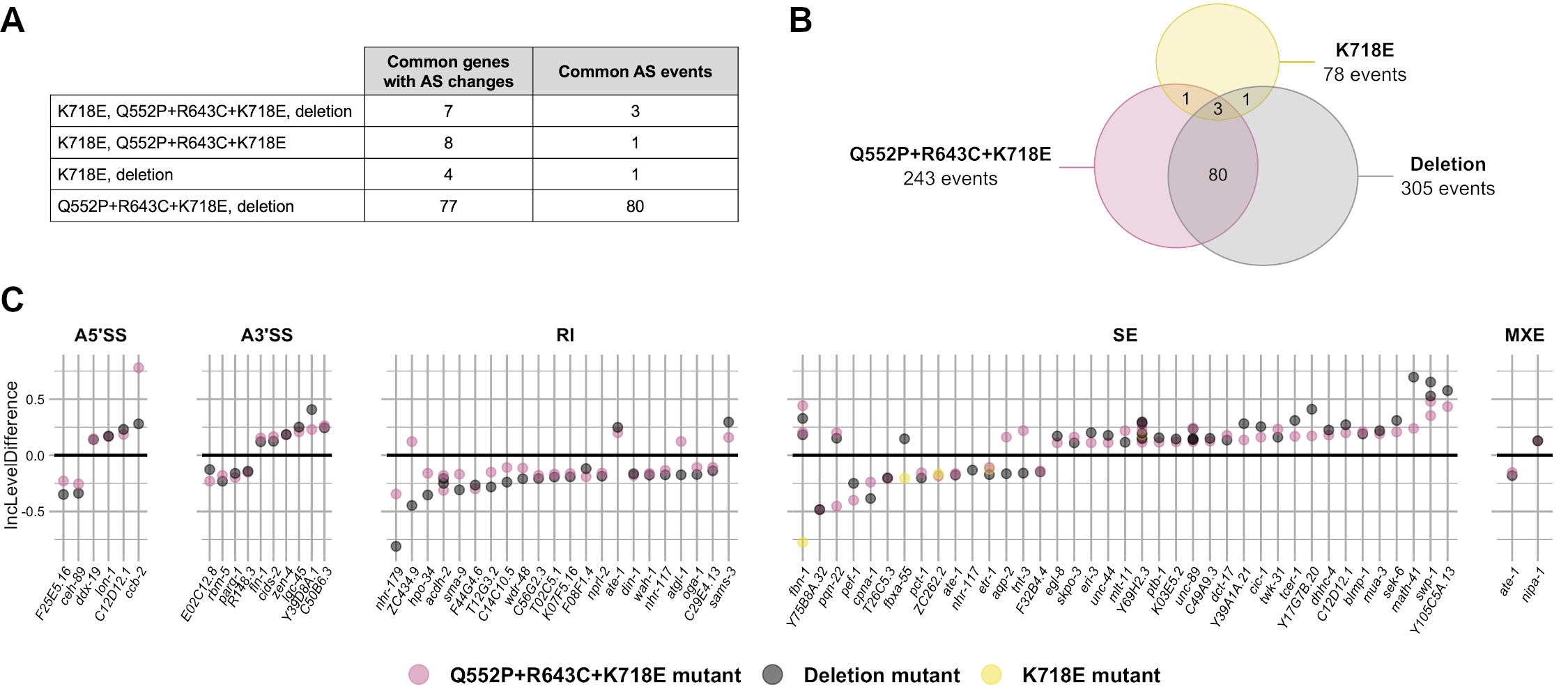

Supplement: S5 Fig — (A) Summary of the overlapping of genes with AS changes (left) and the overlapping of AS events (right) between groups. (B) Venn diagram displaying the overlapping AS events between the three datasets. The total number of significant events with inclusion level difference > 0.1 in each dataset is indicated. (C) Plot showing the inclusion level difference (InclLevelDifference) of AS events that were deregulated in at least two datasets (‘common AS events’), color-coded according to the dataset. Different genes are represented along the x-axis, and events are separated by event type. Some genes (acdh-2, fbn-1, Y69H2.3, unc-89, swp-1, and ate-1) shared more than one event in different datasets. (TIF) [file pgen.1008464.s005.tif]

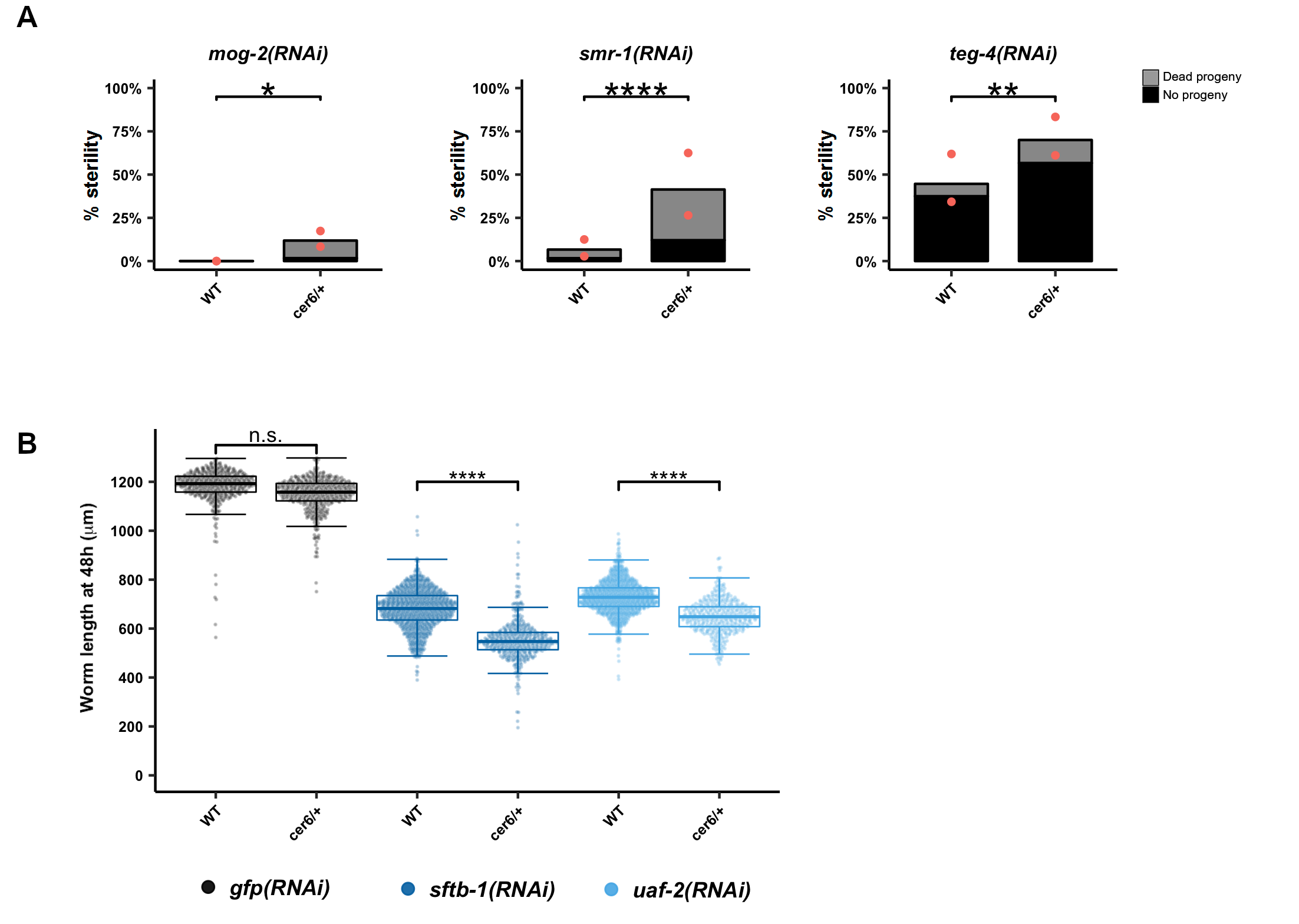

Supplement: S6 Fig — (A) mog-2(RNAi), smr-1(RNAi), and teg-4(RNAi) cause a significant mild increase in sterility in sftb-1(cer6) heterozygotes compared to WT at 25°C (n≥56; N = 2; * p<0.05, ** p<0.01, **** p<0.0001; Fisher’s exact test). Red dots indicate percent sterility observed in each replicate. Gray bars, P0-treated worms giving rise to <5 F1 larvae and some dead embryos (‘dead progeny’ category); black bars, P0-treated worms that laid neither larvae nor dead embryos (‘no progeny’ category). (B) sftb-1(RNAi) and uaf-2(RNAi) induce an earlier larval arrest in cer6/+ heterozygous worms. Dots represent individual worm lengths after 48 h of RNAi treatment at 25°C, overlaid to Tukey-style boxplots (n≥424; N = 2; **** p<0.0001; Kruskal-Wallis test with Dunn’s multiple comparison test). The genotype of the strain used for these experiments was CER190: sftb-1(cer6)/dpy-17(e164) unc-79(e1068) III. (TIF) [file pgen.1008464.s006.tif]

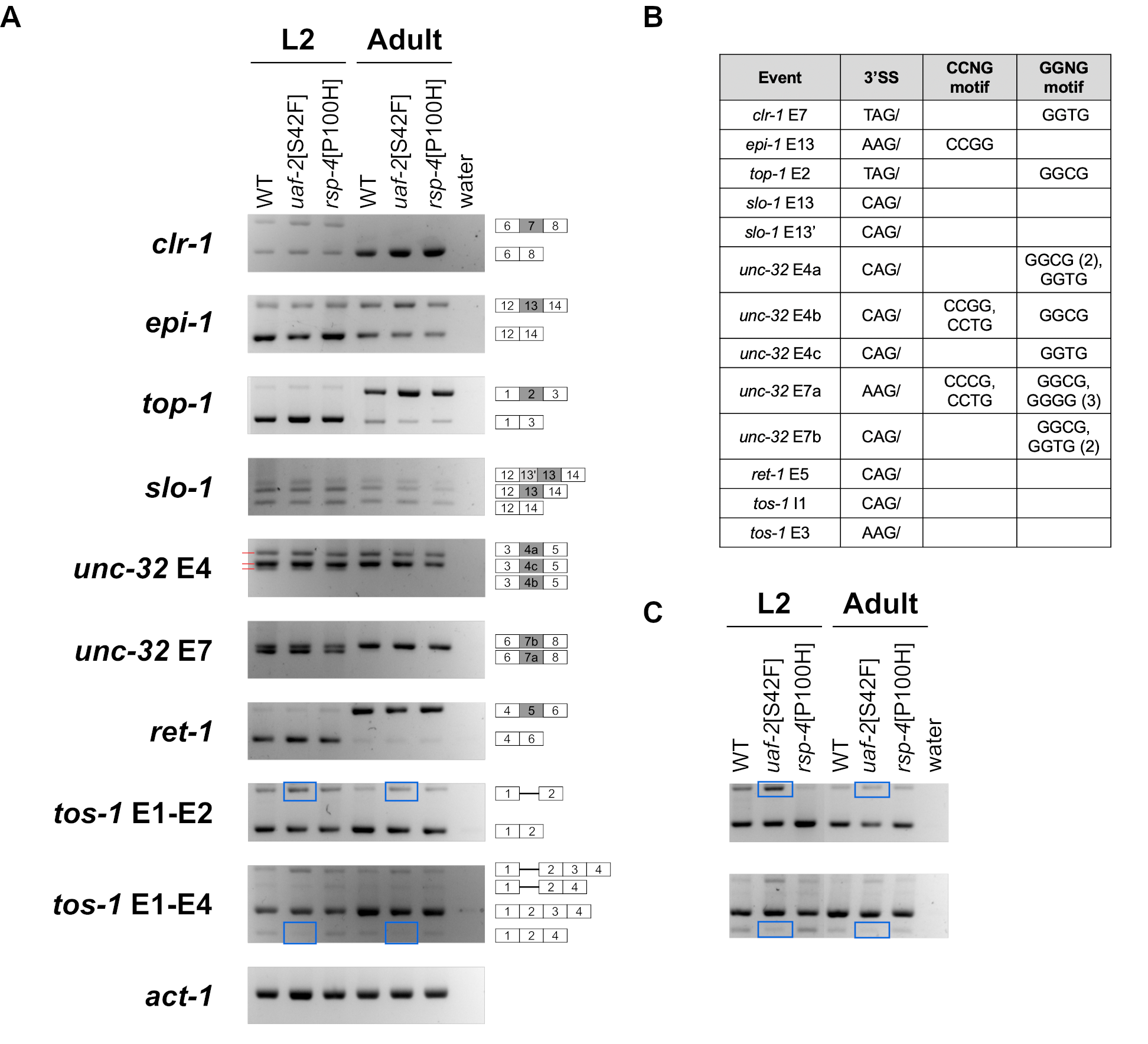

Supplement: S7 Fig — (A) cDNA samples from WT (N2), uaf-2[S42F], and rsp-4[P100H] worms at L2 and adult stage were used to analyze the presence of alternative isoforms by semiquantitative (sq) RT-PCR (N = 1). The alternative exons are numbered and shaded in gray in the schematic representation on the right, together with upstream and downstream exons where the forward and reverse primers annealed, respectively. The three distinct isoforms in unc-32 E4 are indicated with red lines on the left. act-1 was used as an endogenous control. (B) The 3’SS sequence of the alternative exons is indicated, as well as the CCNG and GGNG motifs found in alternative exons using FIMO with a p-value < 0.01. (C) Technical replicate of tos-1 E1-E2 and tos-1 E1-E4 sqRT-PCRs to verify that uaf-2[S42F] promotes intron 1 retention (slowest migrating band in both gels) and exon 3 skipping (fastest migrating band in bottom gel), both indicated with blue boxes. ‘E’ indicates exon, while ‘I’ indicates intron. (TIF) [file pgen.1008464.s007.tif]
