# Supplementary material for: CRISPR editing of sftb-1/SF3B1 in Caenorhabditis elegans allows the identification of synthetic interactions with cancer-related mutations and the chemical inhibition of splicing
Source: PLoS Genet. 2019 Oct 21;15(10):e1008464. doi: 10.1371/journal.pgen.1008464 (PMC6830814; doi:10.1371/journal.pgen.1008464)
Supplement: Attachment — Submitted filename: Serrat et al_response to Reviewers.pdf [file pgen.1008464.s014.pdf]

Serrat et al, 2019. **RESPONSE TO REVIEWERS**

Reviewer #1:

R1-This is an interesting and well-performed study generating several models of cancer-associated spliceosomal gene mutations in C. elegans and identifying genetic and pharmacologic synthetic lethal interactions in these models. The studies are novel and experiments are well performed. Nonetheless, the addition of a few experiments aimed at getting deeper into mechanistic basis for some of the associations shown would help the manuscript. In addition, there are some improper references and citations that should be corrected as noted below:

Authors: We are glad to hear that Reviewer 1 found our study "interesting and well-performed", and appreciate his/her suggestions and comments, which are addressed below.

R1-1.What is the basis for the synthetic lethal effects of mutations in SF3B1 and U2AF1/SRSF2 found here? While this synthetic lethal interaction has been shown in mice, identifying a molecular basis for this interaction was challenging and not very clear.

Authors: The precise molecular basis for these interactions may vary depending on the cell type, as the molecular effects of SF3B1 mutations differ among distinct cancer types. In the case of our model, the synthetic interaction is produced in germ cells and causes sterility. Among the sterile animals, we observed the Mog (masculinization-of-germline) and Tum (tumorous germline) phenotypes, which are hallmarks of general splicing defects during germline development (Kerins *et al*., 2010). During the development of the hermaphroditic germline, RNAi of most of the essential splicing-related genes cause Mog or Tum phenotypes because of a blockage in the sperm-to-oocyte switch or the mitosis-to-meiosis switch, respectively (Kerins *et al*., 2010).

Thus, the genetic interaction between *sftb-1(cer-39)* and *uaf-2* and *rsp-4* mutants could be the consequence of a general alteration in the activity of the spliceosome.

The synthetic interaction between *sftb-1(cer-39)* and *uaf-2*[S42F] is much stronger than with *rsp-4*[P100H]. This result makes sense since U2AF1 has a core function recognizing the 3'SS, whereas SRSF2 is an auxiliary SR protein with less "functional weight" in central splicing processes. Thus, the stronger interaction with *uaf-2*[S42F] may support the hypothesis that these synthetic interactions affect common or core splicing steps.

The main point of this experiment was to underscore the value of our model to investigate vulnerabilities of cells with hampered SF3B1 activity. Still, we understand and share the curiosity of Reviewer 1 for the detailed effect of these genetic interactions at molecular levels, and in the future, we may continue the research line by doing deep RNA-sequencing of different double mutants (synthetic interactions), at different stages and with a very good coverage to detect rare transcripts.

This point is now mentioned in the text, in the results section, page 13, as follows:

"As a result of these synthetic interactions, we observed masculinization of the germline (Mog) and tumorous germline (Tum) phenotypes, which are hallmarks of germlines with altered splicing activity [34]. Thus, these synthetic interactions may cause a synergistic disruption of general splicing functions, perhaps through a flaw in the spliceosome architecture, although detailed information of the molecular basis of this interaction should be obtained by deep sequencing of mutant transcriptomes."

..and in the author summary as follows:

"However, the combination of these mutations has additive effects, hampering SFTB-1 functions and allowing the study of synthetic interactions that may result in a defective spliceosome structure."

R1-2.-Why are the humanized SF3B1 mutant worms sensitive to Pladienolide B but insensitive to Sudemycin D6? These compounds physically interact with several components of the SF3b complex (not just SF3B1). For example, several amino acid residues in PHF5A are required for drug binding to Pladienolide B. It would be important to note whether these proteins are conserved in sequence between C. elegans and humans.

Authors: We understand the curiosity of Reviewer 1 about this point since PB and Sudemycin D6 are molecules with structural similarities. Both molecules have a common pharmacophore (a central diene) but differ in the flanking functional groups. Another difference between the two molecules is that PB is a natural product (derived from bacteria) whereas sudemycin D6 is chemically synthetized. To explore whether the sensitivity to PB was something exceptional, we tested herboxidiene (HB), also known as GEX1A, which is another natural splicing modulator. *C. elegans* with humanized SFTB-1 HEAT repeat 15 was also sensitive to HB and display a dose-response curve when exposed to HB concentration from 1μM to 100 μM. Moreover, in practical terms, this result would be very useful for research laboratories since HB is cheap and commercially available. We have added this new experiment as **Fig 5D**.

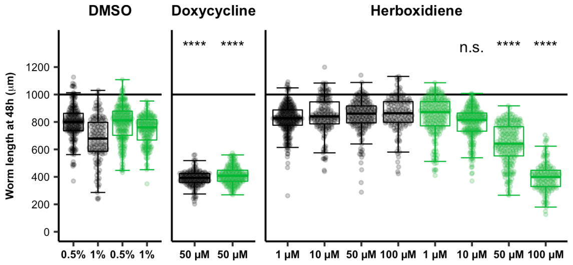

Fig 5D *(D) Worm length after 48h of herboxidiene or doxycycline (control) treatment at 25°C (n≥139; N=3). Data are represented and analyzed as in panel C (n.s., no significant difference).*

This new result is now mentioned in the abstract, author summary, results, and discussion as follows:

Abstract

"Finally, we have edited an SFTB-1 domain to sensitize C. elegans to the splicing modulators pladienolide B or herboxidiene."

Author summary, page 3

"We have edited SFTB-1 to sensitize it to PB and herboxidiene (HB), therefore making C. elegans suitable for assaying the toxicity and efficacy of their chemical derivatives."

"Besides that, we have generated the first C. elegans strain in which splicing can be chemically modulated by PB and HB."

Introduction, page 5

"Thus, we have created the first C. elegans strain sensitive to PB and HB, which would facilitate the chemical modulation of splicing in this model system."

Results, page 14

"In addition, humanization of the HEAT repeat 15 also conferred sensitivity to herboxidiene (HB) (also known as GEX1A) (Fig 5D), which is predicted to be accommodated in the H15-H17 pocket similarly to PB [40,42]. "

Discussion, page 18

"As a result, worms became sensitive to pladienolide B and herboxidiene but maintained resistance to sudemycin D6."

The lack of effect of sudemycin D6 in worms could be due to (i) the SF3B1 domain targeted by sudemycin is not yet completely humanized in *C. elegans* (this could be corrected in the future if we generate a more humanized SFTB-1), or (ii) that sudemycin does not block general splicing and only affects splicing events that are not essential to worm viability. Moreover, as mentioned above, HB and PB are natural compounds, whereas sudemycins are synthetic analogues. Chemically, PB and HB are more similar between them than with sudemycin (Bonnal, Vigevani and Valcárcel, 2012).

Regarding PHF5A and its implication in PB sensitivity, it is true that spliceosome modulators bind to a cavity created by SF3B1 H15-H17 and PHF5A. These two proteins are in direct contact with SF3B3 and SF3B5, and the 4 proteins form the compact SF3B core (Cretu *et al*., 2016, 2018).

PHF5A is highly conserved in *C. elegans* (PHF-5, 91% identity). Two residues in PHF5A have been demonstrated to directly contact PB (Cretu *et al*., 2018) and E7107 (PB derivative) (Finci *et al*., 2018): Y36 and R38. Interestingly, while R38 is conserved, Y36 is a histidine in *C. elegans*. This is surprising because mutation of Y36 to cysteine provides resistance to E7107 (pladienolide derivative), Herboxidiene, Spliceostatin A, and sudemycin D6 (Teng *et al*., 2017).

Tyrosine (Y) has an aromatic hydrophobic side chain, while histidine (H) is a basic residue with a positively charged side chain. Substitution of Y36 to another positively charged polar residue (arginine) provided moderate resistance to E7107 and herboxidiene (Teng *et al*., 2017), suggesting that Y36H would have a similar effect. However, worms humanized for SF3B1 HR15, without Y36 in the PHF5A ortholog, are sensitive to PB and HB.

Regarding other components of the SF3B core, *teg-4*/SF3B3 is also highly conserved (61% identity) but the alignment of the human SF3B5 sequence to the *C. elegans* ortholog *moa-2* only covers 22% of the protein sequences, indicating that this small protein might not be as conserved in worms. Thus, it is possible that further humanization of the SF3B core would affect the sensitivity to splicing inhibitors in worms. We have added this point to the discussion, in page 18, as follows:

"Further humanization of SFTB-1 and other SF3B complex components would likely increase the sensitivity to these drugs and expand the number of known effective splicing modulators in C. elegans. As an example, while PHF5A is highly conserved in C. elegans, the Y36 position, which confers resistance to PB, HB, and sudemycin D6 if mutated (Teng *et al.*, 2017)*, corresponds to a histidine. Thus, the H36Y substitution in C. elegans might increase sensitivity to splicing modulators*."

R1-3-What are the effects of mutations in U2AF1S34P and SRSF2P95H on splicing? These mutations have been reported to impact splicing based on exonic sequences and/or sequences at the 3' splice site (genomic regions which may be more conserved across species).

Authors: We share this curiosity with the Reviewer 1. In fact, in the discussion, we proposed to address this point in the future:

"Additionally, C. elegans rsp-4 and uaf-2 missense mutations could be used for RNA-seq analyses and RNAi screens to study the consequences and vulnerabilities of these mutations, respectively".

Although an in-depth study would require RNA-seq with good coverage and replicates for accurate quantification, we have explored this question to provide some insights to Reviewer 1 by doing RT-PCRs at two different developmental stages. Thus, we performed RT-PCR for 9 alternative splicing (AS) events in WT, and *rsp-4* and *uaf-2* mutants, in L2 and adult animals. These AS events were selected from previous publications and comprise developmentally regulated AS (Ramani, Calarco and Pan, 2011; Wani and Kuroyanagi, 2017), and AS that were reported to be affected by *uaf-2(RNAi)* (Ma *et al*., 2011; Heintz *et al*., 2017). We only observed a clear change in intron 1 retention and exon 3 skipping of *tos-1*, which are AS events that were affected by *uaf-2(RNAi)* according to Ma et al 2011. We have added these RT-PCR experiments as **new Figure S7**.

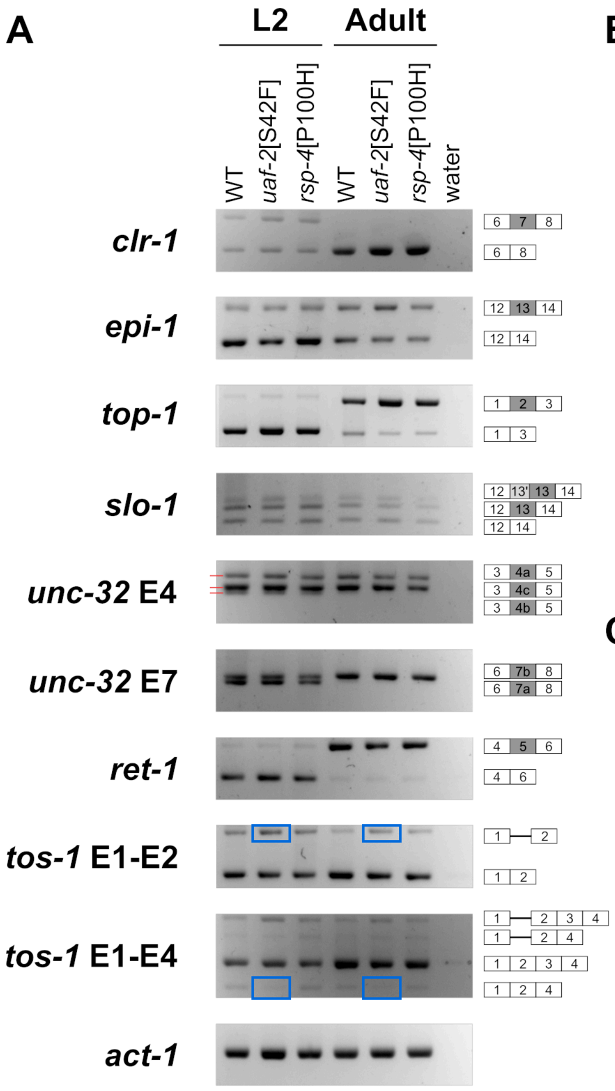

B

Event	3'SS	CCNG motif	GGNG motif
clr-1 E7	TAG/		GGTG
epi-1 E13	AAG/	CCGG	
top-1 E2	TAG/		GGCG
slo-1 E13	CAG/		
slo-1 E13'	CAG/		
unc-32 E4a	CAG/		GGCG (2), GGTG
unc-32 E4b	CAG/	CCGG, CCTG	GGCG
unc-32 E4c	CAG/		GGTG
unc-32 E7a	AAG/	CCCG, CCTG	GGCG, GGGG (3)
unc-32 E7b	CAG/		GGCG, GGTG (2)
ret-1 E5	CAG/		
tos-1 I1	CAG/		
tos-1 E3	AAG/		

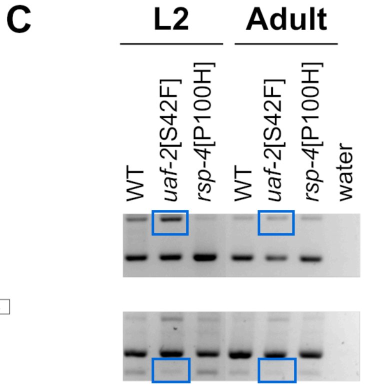

Figure S7. Effect of uaf-2[S42F] and rsp-4[P100H] mutations on a subset of AS events. *(A) cDNA samples from WT (N2), uaf-2[S42F], and rsp-4[P100H] worms at L2 and adult stage were used to analyze the presence of alternative isoforms by semiquantitative (sq) RT-PCR (N=1). The alternative exons are numbered and shaded in gray in the schematic representation on the right, together with upstream and downstream exons where the forward and reverse primers annealed, respectively. The three distinct isoforms in the unc-32 E4 event are indicated with red lines on the left. act-1 was used as an endogenous control. (B) Since human U2AF1* ^{S34F} *affects 3'SS selection and SRSF2* ^{P95H} *promotes the inclusion of exons with CCNG motifs over exons containing GGNG motifs, we looked at these sequences in the studied events. The 3'SS sequence of the alternative exons is indicated, as well as the CCNG and GGNG motifs found in alternative exons using FIMO* (Grant, Bailey and Noble, 2011) *with a p-value < 0.01. (C) Technical replicate of tos-1 E1-E2 and tos-1 E1-E4 sqRT-PCRs to verify that uaf-2[S42F] promotes intron 1 retention (slowest migrating band in both gels) and exon 3 skipping (fastest migrating band in bottom gel), both indicated with blue boxes. 'E' indicates exon, while 'I' indicates intron.*

In the context of the manuscript, we think that this new experiment indicates that *rsp-4* and *uaf-2* mutants do not present dramatic changes in AS regulated events, which is relevant to confirm their synthetic interaction with *sftb-1(cer39)*. This is now stated in the result section, page 13, as follows:

"Neither of these two mutations caused any obvious phenotypes nor dramatic changes in some AS events analyzed by RT-PCR (S7 Fig)."

R1-4-In the Abstract, the following points should be corrected:

R1---SF3B1 mutations have not been shown to impart a growth advantage on their own. In fact, in most studies introduction of mutant SF3B1 actually imparts a growth disadvantage to human and mouse cells. This point alone has been a conundrum related to the study of these mutations.

Authors: It is true that there is a controversy on this point. We have moderated the tone of this sentence in the updated version.

<u>Previous sentence:</u>

"Mutations in SF3B1 confer growth advantages to cancer cells but they may also confer vulnerabilities that can be therapeutically targeted. "

<u>Revised sentence:</u>

"Mutations in SF3B1 likely confer clonal advantages to cancer cells but they may also confer vulnerabilities that can be therapeutically targeted."

R1---Cancer-associated SF3B1 mutations have not been shown to be deleterious when made homozygous in any animal models to date. This has been shown for SRSF2 however (Lee et al. Nature Medice 2016; PMCID PMC4899191). This paper would be important to cite here. SF3B1 mutations have been shown to be lethal when made hemizygous (with ablation of the wild-type allele in the presence of the mutant allele; Zhou et al. A chemical genetics approach for the functional assessment of novel cancer genes. Cancer Research 2015). This paper should also be cited.

Authors: We do not have knowledge about other animal models that are viable with homozygous SF3B1 mutations <u>in the germline</u>, but rather as conditional alleles. In any case, we have removed the beginning of the second sentence from the abstract:

"*~~In contrast to other animal models~~*,..".

We found that the referred paper is Lee et al, 2018 instead of Lee et al, 2016. The reference is now cited in the results section, page 13, as follows:

"In contrast to its murine counterpart SRSF2[P95], rsp-4[P100H] is not deleterious in homozygosis (Lee *et al.*, 2018)*."*

We have now cited Zhou et al, in the discussion, page 15, in the following sentence:

"Cancer cells bear SF3B1 hotspot mutations in heterozygosis and depend on SF3B1 WT function for viability (Zhou *et al.*, 2015)*."*

R1-5-In the Author Summary, the following points should be corrected:

R1---The phrase "visual phenotype" should be edited to be more precise. I believe the authors mean "overt" phenotype (as opposed to a phenotype related to vision) but this needs to be clearly stated.

Authors: "visual" has been replaced with "overt".

---The term "slight" when referencing alterations in splicing should be deleted. First, the splicing of one essential gene in a deleterious way can have massive effect on cells/organisms so the phrase "slight" is not appropriate.

Authors: "only slight" has been replaced with "few".

---Pladienolide B binds to both wild-type SF3B1 protein as well as cancer-associated SF3B1 mutants. Thus, the phrase "hit the wild-type copy" is not appropriate ("copy" also suggests the genomic allele and not the protein).

Authors: "copy" has been replaced with "and the mutated protein".

"..The most promising therapeutic venue is based on pladienolide B (PB) derivatives that hit the wild-type and the mutated protein."

R1-6-The comment in the Discussion about effects of SF3B1 K700E mutations on splicing in mouse versus human cells should note the findings from Lee et al. 2018 reference cited here where a much greater overlap in aberrant splicing events between mouse and human SF3B1 K700E mutant cells was identified than in Obeng et al. or Mupo et. al papers listed (Obeng & Mupo et al. studies missed some very clearly overlapping splicing events across mouse & human SF3B1 K700E mutant cells).

Authors:

The previous sentence

"In these models, the splicing alterations caused by Sf3b1$^{K700E/+}$ were similar to that reported in human tumors, but most of the affected transcripts were different, probably due to low conservation in intronic sequences."

..has been substituted, at page 16, by

"Still, Sf3b1$^{K700E/+}$ mice models only partially recapitulate the hematopoietic phenotype characteristic of MDS (Obeng *et al.*, 2016; Mupo *et al.*, 2017) *and CLL* (Yin *et al.*, 2019)*, although a modest overlap in aberrant splicing events between Sf3b1$^{K700E/+}$ mice and MDS patients with SF3B1 mutations was detected* (Lee *et al.*, 2018)*."*

Reviewer #2:

In this manuscript, Serrat and colleagues design a series of transgenic C. elegans animals carrying cancer-associated mutations in several orthologs of splicing factor genes. The majority of the paper focuses on mutations in sftb-1, the worm ortholog of SF3B1, but they also study mutations introduced in uaf-2/U2AF1 and rsp-4/SRSF2, two additional splicing regulators. The authors have used these transgenic animals to look at phenotypes associated with these cancer-associated mutations. They demonstrate that although individual mutations in sftb-1 do not have dramatic phenotypic effects (unlike in humans), combining several mutations at the same locus do have pronounced effects on animal health and physiology. The authors then explore transcriptome changes in several of these mutant strains by RNA-seq analysis, and find that splicing patterns and gene expression patterns differ between mutants, with severity of phenotype correlating with the extent of transcriptome alterations. Next, the authors perform a focused RNAi screen to look at genetic interactions between alleles of sftb-1 and other splicing related factors, and identify 27 candidate modifier genes and validate one of these interactions with a partial loss of function allele. The authors extend their analysis of genetic interactions by making double mutant animals with other splicing factor genes containing cancer-associated mutations (sftb-1; uaf-2 and sftb-1; rsp-4), and find synthetic interactions between these alleles as well. Finally, the authors also test the responsiveness of a partially humanized version of sftb-1 to pladienolide B, and demonstrate that these transgenic animals display a dose-sensitive phenotype.

Overall, I found that this manuscript has several very interesting results and conceptual advances, including the establishment of a worm strain where splicing can be modulated by a chemical. Although the authors do not delve deeper into any of the interesting genetic interactions uncovered or further pursue experiments related to their humanized sftb-1 mutant that responds to PB, the work presented will pave the way towards additional interesting lines of inquiry. As such I think the work is suitable for publication, but it would be nice to have more clarification/additional data included to strengthen a few points:

Authors: Thanks to Reviewer 2 for his/her precise summary and favorable comments.

R2- 1) In the RNAi screen, it appears that among the 27 hits, the authors score an interaction as more severe relative to a sibling matched wild type from their outcrossing experiments (Table S3). While I appreciate that the authors are trying to use matched sibling wild type strains to account for background effects, the differences in the phenotypes scored for the wild type strains (CER221, CER237, and CER239) can be somewhat variable between experiments. This variability leads to some concerns that there may be variability in the depletion of the genes targeted by RNAi from experiment to experiment rather than variability due to background genetic modifiers (I would be very surprised to see this much variability due to off-target effects due to CRISPR editing for example).

Minimally, it would be nice to see more information on the details of the RNAi screens.

-How many technical/biological replicates were scored.

-Were phenotypes called by different individuals or the same individual
-Were all experiments performed together (i.e same batch of plates / RNAi bacteria) or were different control strains tested on different days?
-This information would give the reader a bit more clarity in terms of how much confidence to place in some of the preliminary RNAi screen data. It is nice that the teg-4 example validates well by additional genetic analysis.

Authors: We have a wide experience in performing RNAi screens. In fact, the PI led the first genome-wide RNAi screen, and synthetic lethality screen with *lin-35* mutants, using the Vidal or ORFeome Library (Rual and Cerón *et al*., 2004). In our experience, RNAi of essential genes (as those related to splicing) and tested in small plates (24-well plates) tend to give noisy results. Synthetic lethal screens with RNAi clones that produces strong phenotypes by itself are challenging. To compensate for these difficulties, our screen included replicates and was repeated by a different person for two of the three mutant backgrounds (**Table S3**).

All assays included two technical replicates (duplicates). In the case of *sftb-1*[K718E] and *sftb-1*[R643C] we performed a biological replicate (the screen was performed twice). The screen in *sftb-1*[Q552P] was performed just once.

The biological replicate in *sftb-1*[K718E] and *sftb-1*[R643C] background was performed by a different person, in a different time. Results from both screens are detailed in Table S3. Sometimes, we did not observe the same phenotypes, probably due to the noisy nature of this screen. We selected a gene as candidate if its RNAi clone produced a clear interaction in at least one of the biological replicates. To clarify the screen, in the **updated Table S3**, we have labeled the different or similar phenotypes observed by one or two persons, in different screens.

The biological replicates were performed on different days, with different batches of plates and RNAi clones, and scored by two different persons (A and B, in updated **Table S3**).

Possible hits	
A	Hit identified in a first RNAi screen performed in K718E and R643C mutant backgrounds, by scientist A.
B	Hit identified in a second RNAi screen performed in K718E, R643C, and Q552P mutant backgrounds, by scientist B.
A, B	Hit identified in both screens

Then, selected candidates were confirmed in additional experiments with bigger plates and, if possible, the ultimate validation of an RNAi screen is to verify the genetic interaction with alleles, as we did for *teg-4*.

We agree with Reviewer 2 that extended information should be provided. Thus, besides the **updated Table S3**, we have now been **extended the corresponding material and method** section as follows:

"Screens in sftb-1[K718E] and sftb-1[R643C] backgrounds were performed twice, at two different times, with different batches of plates and RNAi cultures, and scored by two different persons. Several candidates were selected for further validation, but the existence of false negatives in the screen is possible due to the noisy nature of RNAi for essential genes in small plates."

R2-2) For the transcriptome analysis, it would be nice to know the overlap between the transcriptome changes detected in the different mutants? Either there is a lot of overlap suggesting that the mutations lead to similar effects on sftb-1 activity, or there is not so much overlap suggesting that different mutations have different consequences. Either result is interesting. Perhaps a Venn Diagram or a heatmap would give the reader a better idea of the extent of overlap between targets and direction of change.

Authors: We agree with the Reviewer 2 about this point. The reason for not including this analysis in the previous version was that one of the three transcriptomes was analyzed in a different developmental stage. Still, the overlap between the transcriptome alterations, particularly between the two transcriptomes studied at L2, can be informative. In fact, we have observed a partial overlap between the deregulated transcripts in *sftb-1[Q552P, R643C, K718E]* and *sftb-1(cer6)* (deletion mutant). The deregulated transcripts (coding and non-coding) included in these new Venn diagrams (see below) are significantly deregulated (padj<0.05) and correspond to those plotted in Figure 2C and 2D.

This data is now included as **Figure 2E**. In the new Figure 2, previous panels 2B and 2C that presented redundant information have been merged in a single panel 2B.

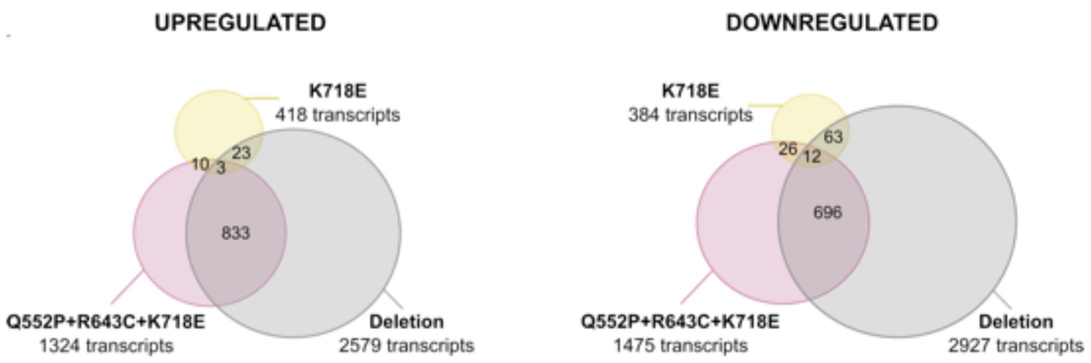

Figure 2E. *Venn diagrams showing the overlap between transcripts that are significantly upregulated (left) or downregulated (right) in the three datasets. The number of transcripts at the intersections is shown, as well as the total number of up- or downregulated transcripts in each mutant strain (padj<0.05).*

The text related to the **new panel 2E** appears in the results section as follows:

*"Interestingly, there is a partial overlap between the transcripts deregulated by the triple mutant allele cer39 and the deletion allele cer6 (**Fig 2E**)... Thus, the additive effect of three missense mutations produces a similar impact on gene expression than the deletion allele but a considerable number of unique AS changes."*

There is little overlap with *sftb-1[K718E]* but this could be due to two reasons: (i) *sftb-1[K718E]* RNA-seq was performed in a different stage (L4) and (ii) *sftb-1[K718E]* deregulated a minor number of transcripts.

R2-3) Are the target transcripts affected by these sftb-1 mutations enriched in any functional categories? Have the authors performed a GO analysis? It would be interesting to hear the results of such an analysis.

Authors: We performed GO terms enrichment analyses, which have been added as a new supplementary figure (**Figure S4**). The results are mentioned in the new version, in the results section, page 9, as follows:

"A functional enrichment analysis uncovered biological pathways that were significantly affected by the distinct sftb-1 alleles (S4 Fig). The K718E mutation caused the downregulation of transcripts related to mitochondrial functions, which were also downregulated by the deletion allele cer6. Both cer39 and cer6 alleles induced the upregulation of transcripts involved in mRNA processing, and the downregulation of different metabolic pathways. Thus, it seems that the common reaction to impaired splicing is to increase the splicing components and reduce metabolism."

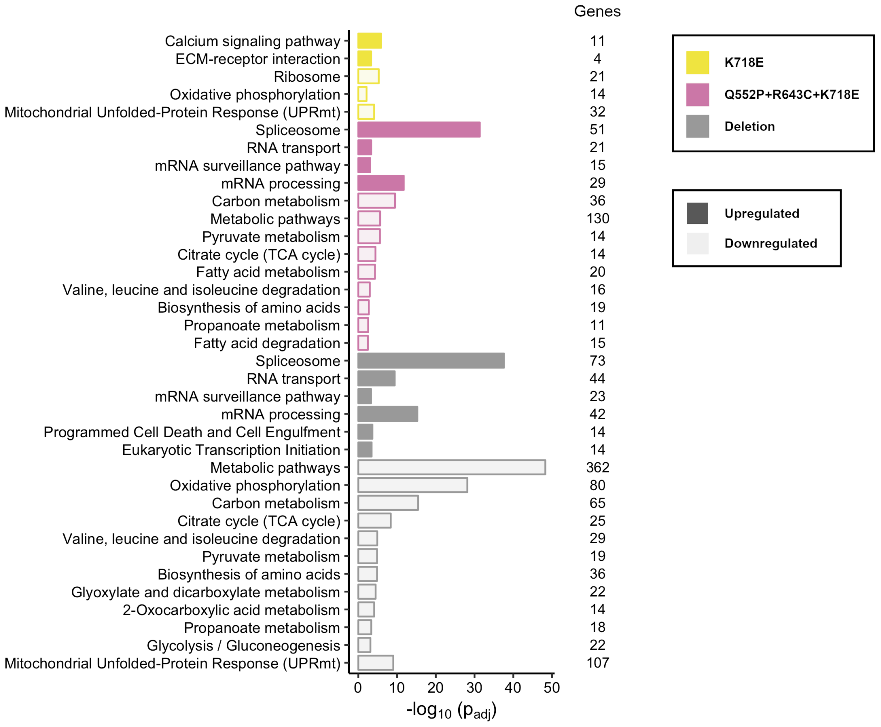

Figure S4. Pathway enrichment analysis of genes with differentially expressed transcripts. *Significantly enriched functional terms in the distinct datasets are listed (padj < 0.01). Bars represent the adjusted enrichment p-values in negative log_{10} scale, color-coded by mutant strain. Solid and clear bars denote enriched terms in upregulated and downregulated genes, respectively. The number of genes with differentially expressed transcripts belonging to each category is shown. The analysis was performed with the g:GOSt tool in g:Profiler* (Raudvere *et al.*, 2019)*, and only biological pathways from KEGG and WikiPathways databases are shown.*

Reviewer #3:

The manuscript by Serrat et al. entitled "CRISPR editing of sftb1/SF3B1 in C. elegans allows the identification of synthetic interactions with cancer-related mutations and the chemical inhibition of splicing." describes the generation and characterization of novel C.elegans mutants in the splicing factor sftb1/SF3B1. The authors describe synthetic lethal interplay between disease-associated sftb1 alleles and mutations in other splicing factors. Moreover, they show that humanizing C.elegans sftb1 sensitizes mutant worms to treatment with the splicing modulator Pladienolide B. Consistent with work in human and murine cells, the authors demonstrate that the disease-associated sftb1 alleles are more sensitive to Pladienolide B treatment. Together, the paper demonstrates the utility of these new animal models to identify novel synthetic lethal interaction with sftb1 mutations. Overall the experiments are sound and well controlled, but a few points need additional clarification.

R3-1. The authors state that the sftb1 loss-of-function mutants are viable until larval stages due to residual maternal contributed wild-type product. Please include quantification of this residual maternal material through embryonic and early larval stages. It is important to understand the requirement for wild-type sftb1 for survival.

Authors: To address this question we made a new strain carrying the deletion allele *sftb-1(cer6)* and the endogenous mCherry reporter for *sftb-1*. Thus, heterozygous animals of this new strain (CER505) have the genotype *sftb-1(cer6/cer114[mCherry::sftb-1])*. Information about this strain has been added to the Material and Methods section as follows:

"To study the duration of maternally inherited SF3B1, we made the strain CER505: *sftb-1(cer6/cer114[mCherry::sftb-1])*."

The results of these experiments are detailed in the new supplementary **Figure S2**, and have been mentioned in the result section, pages 6-7, as follows:

"We formally tested this hypothesis by combining the sftb-1 deletion allele and the sftb-1 endogenous mCherry reporter in the same strain (S2C Figs). The compound heterozygotes produced normal brood size and segregated 19.39% non-red cer6 homozygotes (S2D and S2E Figs). By monitoring mCherry::SFTB-1 expression in the progeny of heterozygous mothers, we observed a small percentage of early L1 larvae with dim red fluorescent expression that later became non-red and developmentally arrested larvae (S2F Fig), indicating that some SFTB-1 maternal product reaches the early L1 stage."

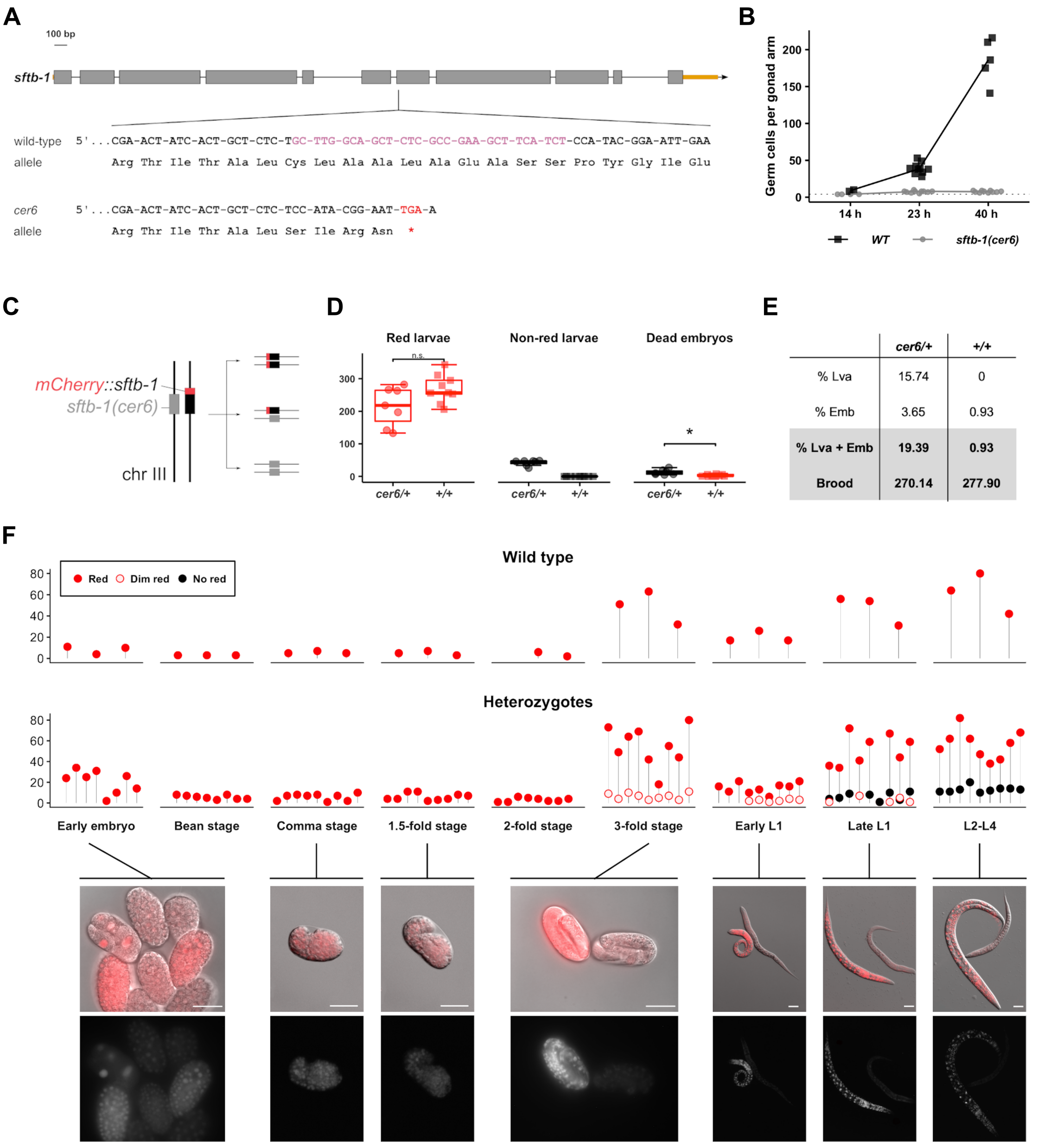

	cer6/+	+/+
% Lva	15.74	0
% Emb	3.65	0.93
**% Lva + Emb**	**19.39**	**0.93**
**Brood**	**270.14**	**277.90**

	3-fold stage	Early L1	Late L1	L2-L4
**% Dim red**	**11%**	**10.3%**	**3.3%**	**0%**
**% No red**	**0%**	**0%**	**14.4%**	**19.2%**

S2 Fig. *sftb-1(cer6)* homozygotes inherit SFTB-1 maternal product.

(A) Schematic representation of the sftb-1 locus. Gray boxes indicate exons, connecting lines indicate introns. *Left* and right orange boxes represent predicted 5’ and 3’ untranslated regions, respectively. In pink are the 29 nucleotides that are deleted in the cer6 allele. Predicted amino acids resulting from translating the WT or cer6 alleles are shown below the DNA sequence. The frameshift caused by the deletion introduces a premature stop codon in the cer6 transcript (colored in red). Scale bar, 100 base pairs (bp). (B) Germ cell number of WT (N2 strain) or homozygous *sftb-1(cer6)* synchronized larvae grown for 14, 23, and 40 hours at 20◦C. In WT worms, differentiating spermatocytes were not included at the last time point, and the number of germ cells was counted in one gonad arm only. Black and gray lines connect the median germ cell number at each time point in WT and *sftb 1(cer6)* worms, respectively. The dotted line represents the four germ cells that are normally present in recently hatched

L1 larva; these cells will resume proliferation during the L1 stage. Between 2 and 10 animals per condition were scored (N=1). (C) Schematic representation of the compound heterozygous strain CER505: *sftb-1(cer6)/ sftb 1(cer114[mCherry::sftb-1])* III. (D) Total number of progeny laid by heterozygous (*cer6*/+) or WT (+/+) worms from a CER505 population (n=7, 9; N=1). The number of F1 larvae expressing mCherry::SFTB-1 was not significantly different between both groups (n.s.), while cer6/+ animals laid a significantly higher number of dead embryos which lacked red fluorescence (Mann-Whitney's test; * $p<0.05$). Heterozygotes also segregated a number of non-red larvae that were arrested and were not observed in WT plates, indicating that they were cer6/cer6 animals. '+' denotes the *cer114[mCherry::sftb-1*] allele. Dots represent measures in individual worms, overlaid to Tukey-style boxplots. (E) Mean percentage of non-red F1 arrested larvae (% Lva), F1 dead embryos (% Emb), and mean brood size in worms of the indicated genotypes, from data represented in panel D. (F) mCherry::SFTB-1 expression in F1 progeny from wild-type (top, n=3) or heterozygous (bottom, n=9) animals from a CER505 population (N=1). At each stage, vertical lines correspond to individual worms, and solid red, open red, and black dots represent the total number of red, dim red, or non-red progeny observed, respectively. Representative DIC and fluorescence microscopy images are shown below the graph (scale bar, 25 μm). The mean percentage of dim red and non-red progeny laid by heterozygotes is indicated in late stages, and is not specified in early stages as it was 0%.

R3-2. Knockdown of sftb1 was shown to be synthetic lethal with sftb1 (Q55P, R643C, K718E) mutants. Studies in murine models of other splicing factors suggest that cells heterozygous with null alleles are less affected than those heterozygous with disease-associated alleles. It would be of value to the community to know if this is also true for SF3B1. The genetic interactions should also be performed with sftb1 null/wild type worms.

Authors: We performed *sftb-1(RNAi)* in *cer6*/+ worms and found that these heterozygous animals are also sensitive to *sftb-1(RNAi) uaf-2(RNAi*), *mog-2(RNAi)*, *teg-4(RNAi)* and *smr-1(RNAi).* This is now shown as **new Fig S6** and mentioned in the text, page 12, in the results section, as follows:

"We also found that heterozygous animals carrying the deletion allele sftb-1(cer6) were sensitive to RNAi of the five interactors (S6 Fig). Still, double mutant strains are better than cer6 heterozygous animals for identifying genetic interactions (Figs 3 and S6)."

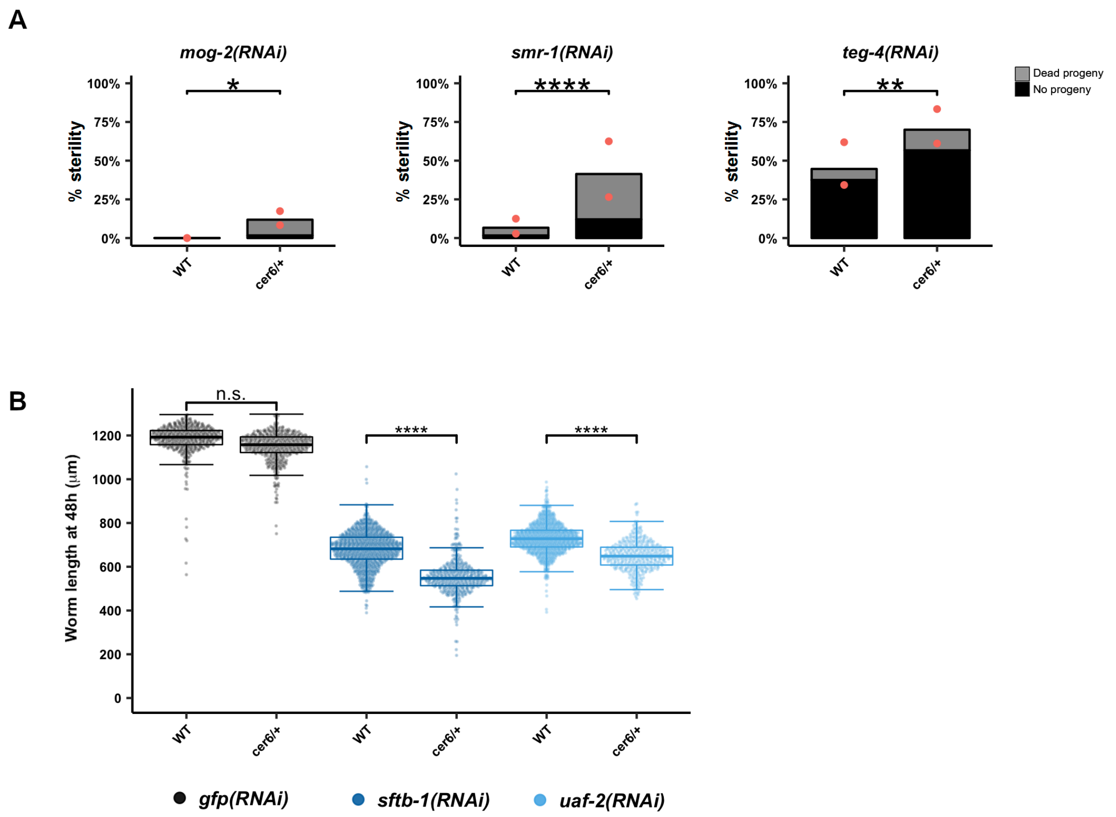

Figure S6. sftb-1(cer6) heterozygotes are sensitive to RNAi of five interactors. *(A) mog-2(RNAi), smr-1(RNAi), and teg-4(RNAi) cause a significant mild increase in sterility in sftb-1(cer6) heterozygotes compared to WT at 25oC ($n \geq 56$; N=2; * $p<0.05$, ** $p<0.01$, **** $p<0.0001$; Fisher's exact test). Red dots indicate percent sterility observed in each replicate. Gray bars, P0-treated worms giving rise to <5 F1larvae and some dead embryos ('dead progeny' category); black bars, P0-treated worms that laid neither larvae nor dead embryos ('no progeny' category). (B)sftb-1(RNAi) and uaf-2(RNAi) induce earlier larval arrest in cer6/+heterozygous worms. Dots represent individual worm length after 48 h of RNAi treatment at 25oC, overlaid to Tukey-style boxplots ($n \geq 424$; N=2; **** $p<0.0001$; Kruskal-Wallis test with Dunn's multiple comparisons test). The genotype of the strain used for these experiments was CER190: sftb-1(cer6)/dpy-17(e164) unc-79(e1068) III.*

R3-3. There are similar types of splicing changes in the various sftb1 mutants, but are the same transcripts/genes affected. Quantify the overlap of the exact splicing changes across the RNA-seq datasets and add 1-2 sentences commenting on the overlaps.

Authors: Reviewer 2 made a similar request in his/her point 2, which was addressed by adding the new figure 2E. In addition, reviewer 3 asked for the "exact splicing changes". To satisfy this request we have made a new supplementary figure, Fig S5, including the genes presenting an altered alternative splicing in the different mutants, which is referred to in the text, page 9, as follows:

"We also found few AS events that are similarly altered in distinct sftb-1 mutants. The largest overlap was again observed between the cer39 and the cer6 alleles, which shared 80 deregulated events (S5 Fig)."

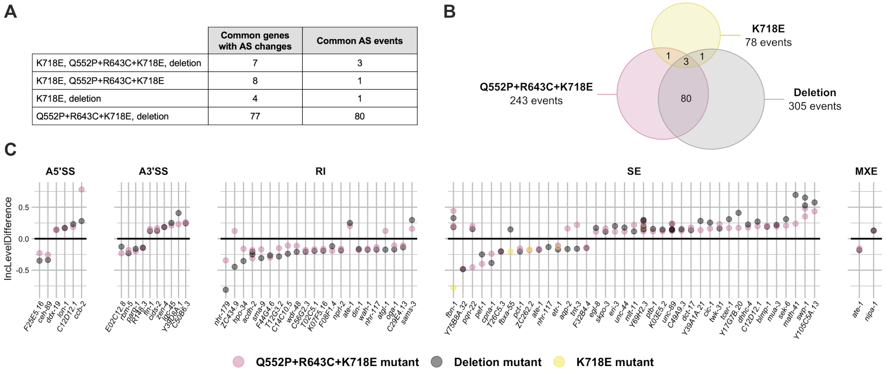

Figure S5. Common AS events deregulated by sftb-1 alleles. *(A) Summary of the overlapping of genes with AS changes (left) and the overlapping of AS events (right) between groups. (B) Venn diagram displaying the overlapping AS events between the three datasets. The total number of significant events with inclusion level difference > 0.1 in each dataset is indicated. (C) Plot showing the inclusion level difference (InclLevelDifference) of AS events that were deregulated in at least two datasets ('common AS events'), color-coded according to the dataset. Different genes are represented along the x axis, and events are separated by event type. Some genes (acdh-2, fbn-1, Y69H2.3, unc-89, swp-1, and ate-1) shared more than one event in different datasets.*

R3-4. Out of the 104 RNAi clones tested, 27 showed a synthetic interaction with the sftb1 mutants. Is there anything special about the 27 compared to the 104? Do they act as a specific step of the splicing cycle? Are they enriched for SF3B1-interacting proteins? Please add 1-2 sentences commenting on the hits.

Authors: Information about the 27 candidates is included in Table S3, but we have included this information as a sentence is the main text, page 10, as follows:

"This led us to identify 27 splicing factors whose knockdown phenotype was enhanced in at least one of the three *sftb-1* single mutants, suggesting possible synthetic interactions. Among the potential candidates, we identified proteins present in pre-catalytic and catalytic spliceosomes, as well as a few auxiliary factors, indicating that *sftb-1* missense mutations might impact other steps of the splicing reaction besides early spliceosome assembly (S3 table)."

R3-5. RNAi for teg4 is the strongest interactor with the sftb1 mutants, but it also shows major lethality in wild types. Add a sentence clarifying the effect is not overly selective to the sftb1 mutants.

Authors: This point has now been clarified by modifying the previous sentence, on page 11, as follows:

Previous sentence:

"Finally, we further validated the synthetic interaction between *teg-4* and *sftb-1,* as *sftb-1* double mutants were more sensitive to *teg-4(RNAi)* (**Fig 3G**)."

Updated sentence:

"Finally, *sftb-1* double mutants were more sensitive to *teg-4(RNAi)* (**Fig 3G**) This former synthetic interaction was not as strong as in *mog-2(RNAi)* and *smr-1(RNAi)* but was supported by the genetic interaction between genetic alleles (**Fig 3B**)."

R3-6. By mutating four amino acids in sftb1, the authors were able to sensitize worms to pladienolide B but not sudemycin D6. Please add a sentence to the results or discussion commenting on the different binding sites for PB and sudemycin within SF3B1 that imparted this specificity.

Authors: Reviewer 1, in his/her point 2, did a similar request (see above).

PB and Sudemycin D6 have a common pharmacophore (a central diene), but differ in flanking functional groups. Another difference between the two molecules is that PB is a natural product (derived from bacteria) whereas sudemycin D6 is chemically synthetized. To explore whether the sensitivity to PB was something exceptional, we also tested Herboxidiene (HB) (also known as GEX1A), which is another natural splicing modulator. *C. elegans* SFTB-1 with humanized HR15 was also sensitive to HB. We have added this new experiment as **Figure 5D**.

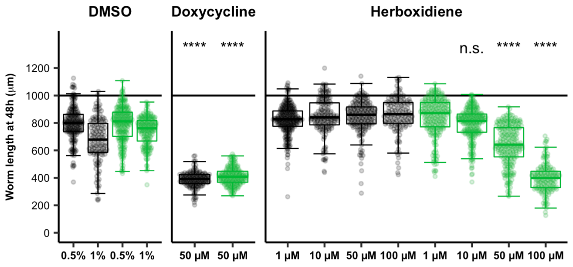

New fig 5D: (D) Worm length after 48h of herboxidiene or doxycycline treatment at 25°C (n≥139; N=3). Wild-type animals are represented as black dots, and *sftb-1(cer144)* mutants as green dots**.** Data are represented and analyzed as in panel C (n.s., no significant differences).

This result, as detailed above (reviewer 1), is now mentioned in the abstract, author summary, results, and discussion.

The lack of effect for sudemycin in worms could be due to (i) the SF3B1 domain targeted by sudemycin is not yet completely humanized in *C. elegans* yet (this could be corrected in the future if we got a more humanized SFTB-1), or that sudemycin does not block general splicing and only affects splicing events that are not essential to worm viability.

Thus, it is possible that further humanization of the SF3B core would affect the sensitivity to splicing inhibitors. We have added this point to the discussion, page 18, as follows:

"Further humanization of SFTB-1 and other SF3B complex components would likely increase the sensitivity to these drugs and expand the number of effective splicing modulators in C. elegans."

Have all data underlying the figures and results presented in the manuscript been provided?

Large-scale datasets should be made available via a public repository as described in the *PLOS Genetics* data availability policy, and numerical data that underlies graphs or summary statistics should be provided in spreadsheet form as supporting information.

Reviewer #1: Yes

Reviewer #2: No: Large-scale transcriptome datasets do not have associated accesssion numbers. Presumably the authors will upload these to the NCBI upon acceptance

We have provide this information as follows:

Data availability

The RNA sequencing data generated in this study have been deposited in the Gene Expression Omnibus repository and are available under the accession number GEO: GSE129642.

Reviewer #3: Yes

List of strains in now included as Table S6
